# MSC-AS1 induced cell growth and inflammatory mediators secretion through sponging miR-142-5p/DDX5 in gastric carcinoma

**DOI:** 10.18632/aging.202800

**Published:** 2021-04-04

**Authors:** Yan Liu, Lin Li, Xiaoxu Wu, Haiyan Qi, Yan Gao, Yanqi Li, Da Chen

**Affiliations:** 1 Department of Oncology, The Fourth Hospital of China Medical University, Liaoning, Shengyang 110032, China; 2 Department of General Practice, The Fourth Hospital of China Medical University, Liaoning, Shengyang 110032, China

**Keywords:** MSC-AS1, miR-142-5p, DDX5, gastric cancer

## Abstract

Emerging studies have noted that dysregulated lncRNAs are implicated in cancer progression and tumorigenesis. We first showed that MSC-AS1 was overexpressed in gastric cancer (GC) cells (HGC-27, MKN-45, SGC-7901 and MGC-803 cells) compared with GES cells. We observed that MSC-AS1 was upregulated in GC specimens compared with paired normal specimens. MSC-AS1 increased cell growth and cycle progression. Moreover, the overexpression of MSC-AS1 enhanced the secretion of the inflammatory mediators IL-1β, IL-6 and TNF-α. We found that the overexpression of MSC-AS1 inhibited the expression of miR-142-5p in HGC-27 cells. We noted that DDK5 was a target gene of miR-142-5p. The overexpression of miR-142-5p suppressed the luciferase activity of wild-type DDX5, but the luciferase activity of the mutant DDX5 was not changed. We showed that miR-142-5p was downregulated in GC specimens compared with paired normal specimens. MSC-AS1 expression was inversely correlated with miR-142-5p expression in GC specimens. MSC-AS1 induced cell growth, cell cycle progression and inflammatory mediator secretion by modulating DDX5. These results showed that MSC-AS1 functions as a key oncogene in the development of GC.

## INTRODUCTION

Gastric cancer (GC) is the 3^rd^ most common cause of tumor-associated death and the 3^rd^ most commonly diagnosed tumor worldwide [1–4]. The cause and pathogenesis of this disease are complex and associated with many factors [5–8]. Despite the great achievements that have been made in GC therapeutics, the survival rate of GC patients remains unsatisfactory [9–12]. The major challenge in the treatment of advanced GC is the manifestation of peritoneal, distal organ and lymphatic metastases [13–15]. Therefore, a detailed and improved understanding of the molecular mechanisms underlying GC progression and development is greatly needed.

As one type of ncRNA, lncRNAs are more than 200 nt long [16–18]. Growing evidence has revealed that lncRNAs are involved in several cellular pathways and processes, including cell apoptosis, angiogenesis, differentiation, immune responses, proliferation and metabolism [19–21]. Compelling studies have shown that lncRNAs play critical roles in the development and initiation of tumors and that lncRNAs act as tumor suppressor genes or oncogenes in tumors [22–25]. Recently, a new lncRNA, MSC-AS1, was identified as a crucial modulator in the development of tumors [26–30]. Cao et al [31]. found that MSC-AS1 promoted hepatocellular carcinoma progression by enhancing PGK1 expression. Yao et al [30]. showed that |MSC-AS1 increased nasopharyngeal carcinoma development by regulating miR-524-5p/NR4A2. However, the functional role of MSC-AS1 in GC remains unknown.

## RESULTS

### MSC-AS1 and DDX5 were overexpressed and miR-142-5p was downregulated in GC cells

**Figure 1:**
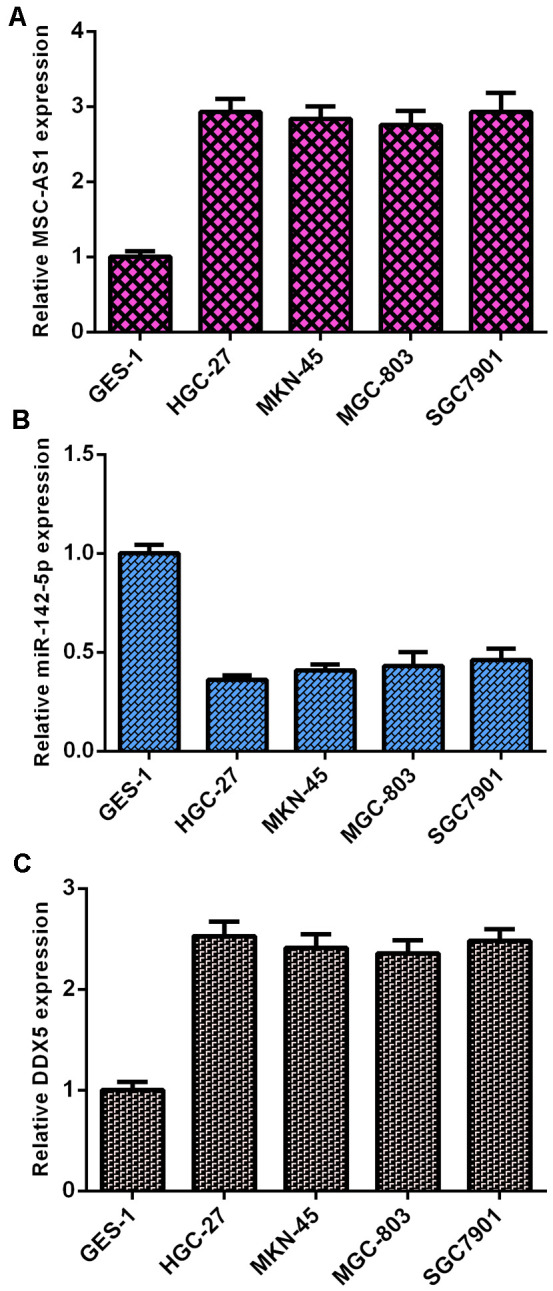
**MSC-AS1 and DDX5 were overexpressed and miR-142-5p was downregulated in GC cells.** (**A**) The expression of MSC-AS1 was detected by qRT-PCR analysis. GAPDH was used as the internal control. (**B**) The expression of miR-142-5p was detected by qRT-PCR analysis. U6 was used as the internal control. (**C**) DDX5 was upregulated in GC cells (HGC-27, MKN-45, SGC-7901 and MGC-803 cells) compared to GES cells. GAPDH was used as the internal control.

MSC-AS1 was overexpressed in GC cells (HGC-27, MKN-45, SGC-7901 and MGC-803 cells) compared to GES cells (Figure 1A). miR-142-5p was downregulated in GC cells (HGC-27, MKN-45, SGC-7901 and MGC-803 cells) compared to GES cells (Figure 1B).

### MSC-AS1 was upregulated in GC specimens

**Figure 2:**
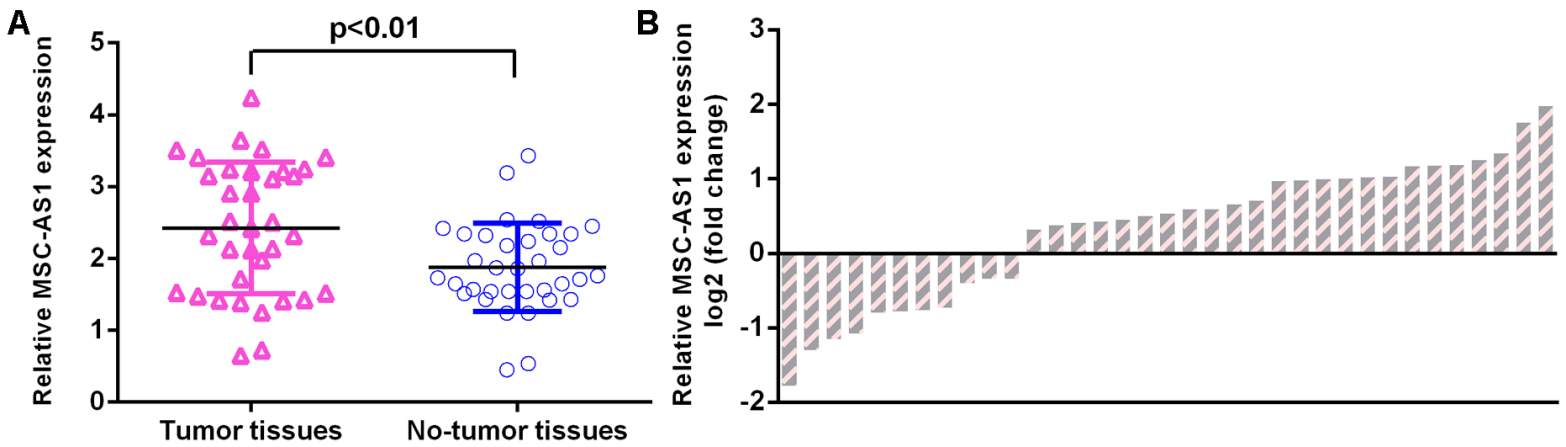
**MSC-AS1 was upregulated in GC specimens.** (**A**) MSC-AS1 was upregulated in GC specimens compared with paired normal specimens. (**B**) The level of MSC-AS1 was upregulated in 29 GC specimens (72.5%, 29/40) compared to their paired normal specimens. GAPDH was used as the internal control.

We observed that MSC-AS1 was upregulated in GC specimens compared with paired normal specimens (Figure 2A). The level of MSC-AS1 was upregulated in 29 GC specimens (72.5%, 29/40) compared to their paired normal specimens (Figure 2B).

### miR-142-5p was downregulated in GC specimens

**Figure 3:**
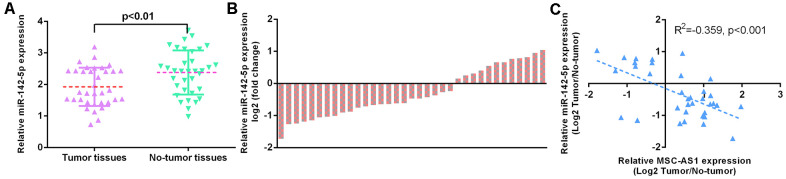
**miR-142-5p was downregulated in GC specimens.** (**A**) miR-142-5p was downregulated in GC specimens compared to paired normal specimens. (**B**) The level of miR-142-5p was decreased in 28 GC specimens (70.0%, 28/40) compared to their paired normal specimens. (**C**) MSC-AS1 expression was inversely correlated with miR-142-5p expression in GC specimens. U6 was used as the internal control.

We showed that miR-142-5p was downregulated in GC specimens compared to paired normal specimens (Figure 3A). The level of miR-142-5p was decreased in 28 GC specimens (70.0%, 28/40) compared to their paired normal specimens (Figure 3B). MSC-AS1 expression was inversely correlated with miR-142-5p expression in GC specimens (Figure 3C).

### MSC-AS1 increased cell growth, cell cycle progression and inflammatory mediator secretion

**Figure 4:**
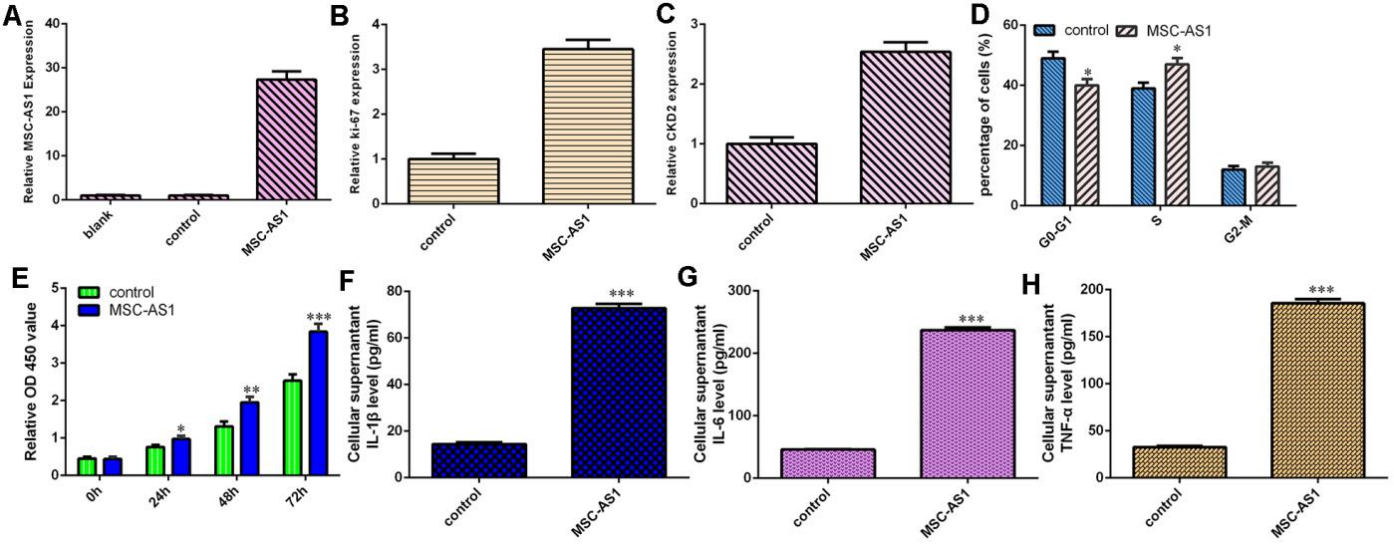
**MSC-AS1 increased cell growth, cell cycle progression and inflammatory mediator secretion.** (**A**) The expression of MSC-AS1 was detected by qRT-PCR analysis. (**B**) The expression of Ki-67 was measured using qRT-PCR analysis. (**C**) The level of CKD2 was detected by qRT-PCR analysis. (**D**) MSC-AS1 overexpression induced cell cycle progression in HGC-27 cells. (**E**) MSC-AS1 overexpression increased HGC-27 cell growth, based on CCK-8 analysis. (**F**) MSC-AS1 overexpression enhanced IL-1β secretion. (**G**) MSC-AS1 overexpression promoted IL-6 secretion. (**H**) Elevated expression of MSC-AS1 increased the secretion of TNF-α. GAPDH was used as the internal control. *p<0.05, **p<0.01 and ***p<0.001.

The level of MSC-AS1 was significantly overexpressed in HGC-27 cells after treatment with the pcDNA-MSC-AS1 plasmid (Figure 4A). Elevated expression of MSC-AS1 enhanced the expression of Ki-67 (Figure 4B) and CKD2 (Figure 4C) in HGC-27 cells. MSC-AS1 overexpression induced cell cycle progression in HGC-27 cells (Figure 4D). Overexpression of MSC-AS1 increased HGC-27 cell growth, according to the CCK-8 analysis (Figure 4E). Overexpression of MSC-AS1 enhanced the secretion of the inflammatory mediators IL-1β, IL-6 and TNF-α (Figure 4F–4H).

### MSC-AS1 modulated miR-142-5p/DDX5 expression

**Figure 5:**
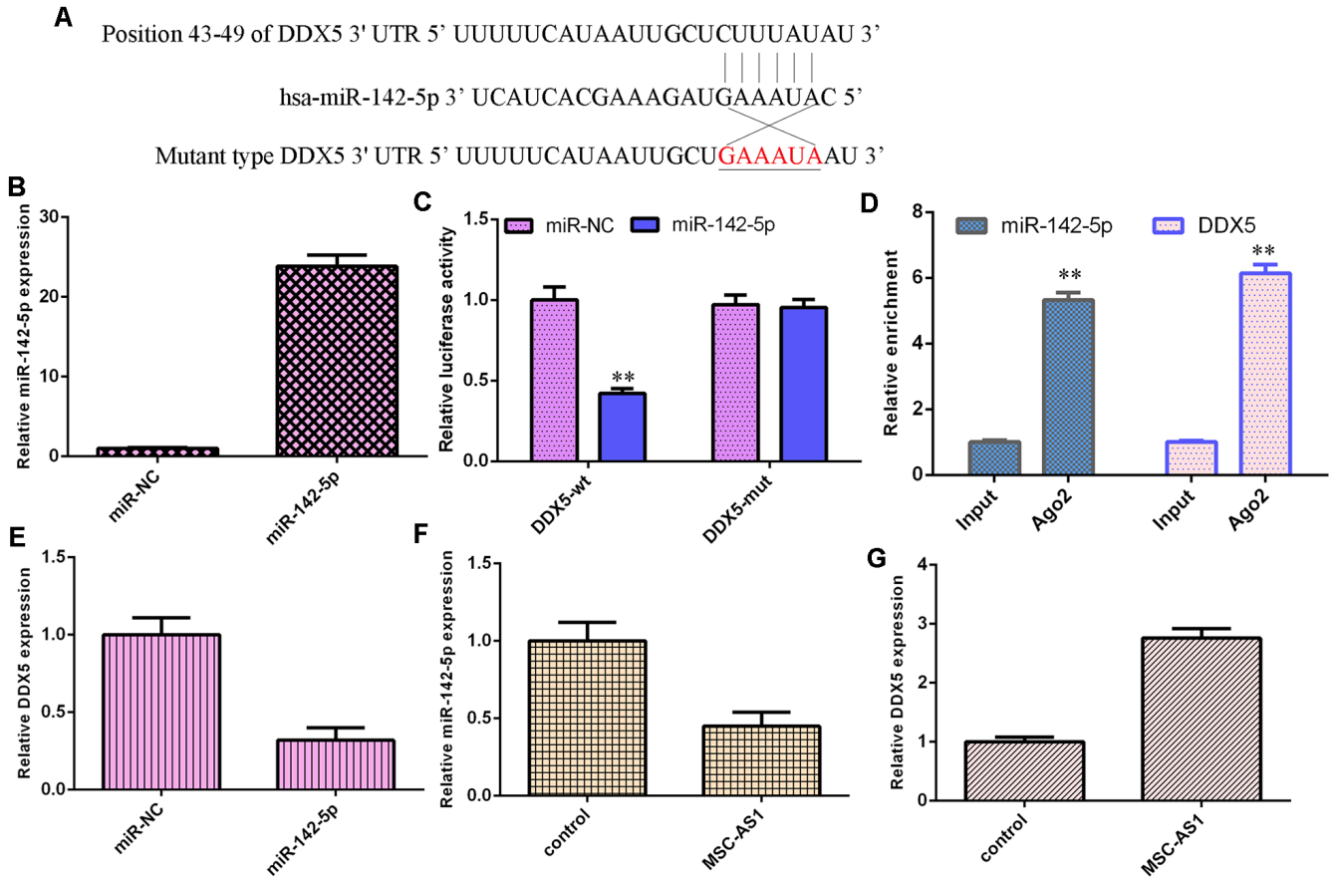
**MSC-AS1 modulated miR-142-5p/DDX5 expression.** (**A**) By utilizing an online tool, TargetScan, DDX5 was predicted to be a potential target gene of miR-142-5p. (**B**) The expression of DDX5 was measured by using qRT-PCR analysis. (**C**) Overexpression of miR-142-5p suppressed the luciferase activity of wild-type DDX5, but the luciferase activity of mutant DDX5 was not changed. (**D**) DDX5 and miR-142-5p were enriched in the Ago2-containing beads compared with the input, according to the RIP method. (**E**) miR-142-5p overexpression decreased DDX5 expression. (**F**) MSC-AS1 overexpression inhibited miR-142-5p expression. (**G**) The expression of DDX5 was measured by qRT-PCR assay. **p<0.01.

By utilizing the online tool TargetScan, DDX5 was predicted to be a potential target gene of miR-142-5p (Figure 5A). DDX5 was significantly overexpressed in HGC-27 cells after treatment with the DDX5 mimic (Figure 5B). Overexpression of miR-142-5p suppressed the luciferase activity of wild-type DDX5, but the luciferase activity of mutant DDX5 was not changed (Figure 5C). Moreover, DDX5 and miR-142-5p were enriched in the Ago2-containing beads compared with the input, according to the RIP method (Figure 5D).

miR-142-5p overexpression decreased DDX5 expression (Figure 5E). MSC-AS1 overexpression inhibited miR-142-5p expression (Figure 5F) and enhanced DDX5 expression in HGC-27 cells (Figure 5G).

### MSC-AS1 induced cell growth, cell cycle progression and inflammatory mediator secretion by modulating DDX5

**Figure 6:**
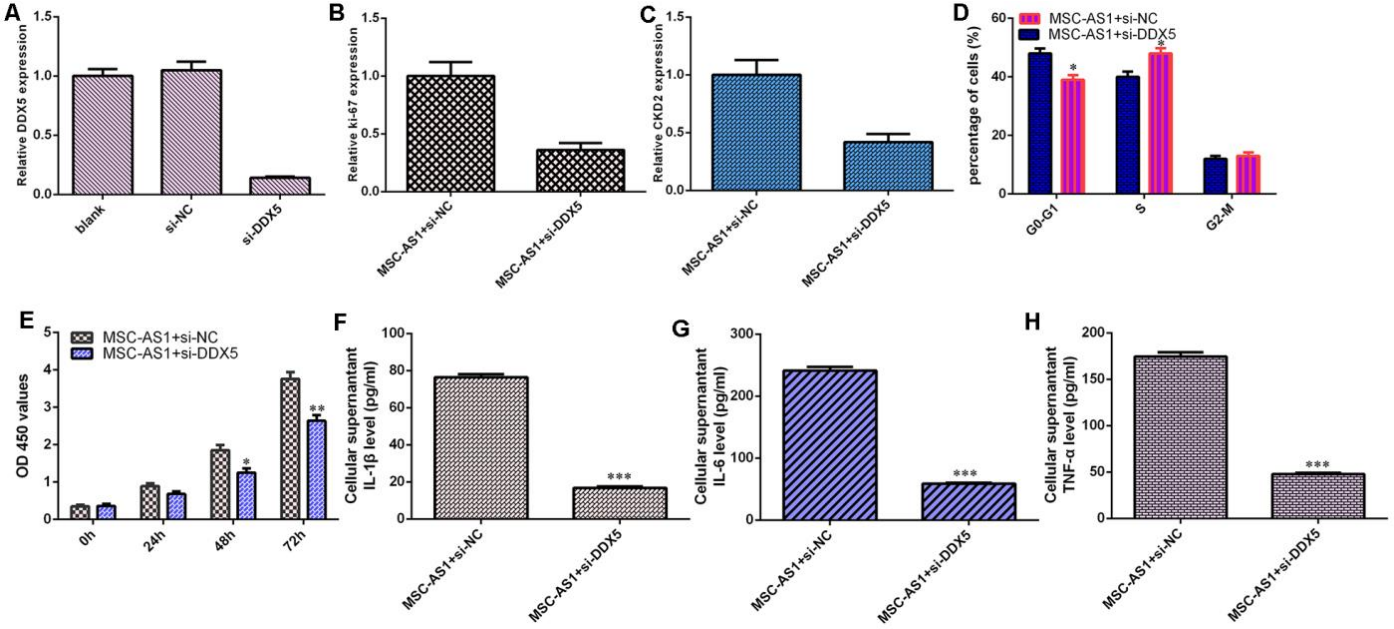
**MSC-AS1 induced cell growth, cycle and inflammatory mediators secretion via modulating DDX5.** (**A**) The level of DDX5 was measured by qRT-PCR analysis. (**B**) The expression of ki-67 was measured by qRT-PCR assay. (**C**) The expression of CKD2 was detected by qRT-PCR assay. (**D**) Inhibition expression of DDX5 decreased cell cycle in MSC-AS1-overexpressing HGC-27 cell. (**E**) Cell proliferation was measured by analyzed with CCK-8 assay. (**F**) The expression of IL-1β was measured by ELISA. (**G**) The expression of IL-6 was measured by ELISA. (**H**) The expression of IL-6 was detected by ELISA. *p<0.05, **p<0.01 and ***p<0.001.

The level of DDX5 was significantly decreased in HGC-27 cells after treatment with DDX5 siRNA (Figure 6A). Knockdown of DDX5 suppressed the expression of Ki-67 (Figure 6B) and CKD2 (Figure 6C) in MSC-AS1-overexpressing HGC-27 cells. Inhibition of DDX5 expression decreased the cell cycle (Figure 6D) and growth (Figure 6F) of MSC-AS1-overexpressing HGC-27 cells. Knockdown of DDX5 suppressed the secretion of the inflammatory mediators IL-1β, IL-6 and TNF-α (Figure 6F–6H).

## DISCUSSION

Compelling studies have observed that lncRNAs participate in many cell biological processes, including carcinogenesis and development [32–34]. For instance, Sun et al [35]. proved that lncRNA LATS2-AS1-001 suppressed GC development by modulating the YAP1/LATS2 pathway by binding to EZH2. Sun et al. indicated that AK025387 promoted cell invasion and migration in GC [36]. Gao et al. observed that NEAT1 induced GC development by modulating the miR-365a-3p/ABCC4 axis. Zhang et al [37]. showed that NNT-AS1 knockdown suppressed GC development by regulating the miR-142-5p/Wnt/β-catenin/SOX4 signaling pathway. Li et al [38]. showed that IGF2-AS enhanced GC cell migration, invasion and growth by regulating the EZH2/miR-937 axis. Recently, a new lncRNA, MSC-AS1, was identified as a crucial modulator in the development of tumors [26–30]. Cao et al [31]. Found that MSC-AS1 enhanced hepatocellular carcinoma progression by enhancing PGK1 expression. Yao et al [30]. showed that MSC-AS1 increased nasopharyngeal carcinoma development by regulating miR-524-5p/NR4A2. Hu et al [28]. showed that MSC-AS1 regulated renal carcinoma cell migration and growth by modulating the miR-3924/WNT5A/Wnt/β-catenin axis. In the present study, we conducted tests to examine the functional role of MSC-AS1 in GC. We first showed that MSC-AS1 was overexpressed in GC cells (HGC-27, MKN-45, SGC-7901 and MGC-803 cells) compared to GES cells. We observed that MSC-AS1 was upregulated in GC specimens compared with paired normal specimens. MSC-AS1 increased cell growth and cell cycle progression. Moreover, the overexpression of MSC-AS1 enhanced the secretion of the inflammatory mediators IL-1β, IL-6 and TNF-α.

Numerous studies have shown that lncRNAs regulate cell biological processes by sponging miRNA expression [39–41]. For example, Deng et al [42]. showed that DLGAP1-AS1 induced GC aggressiveness by sponging miR-628-5p and regulating the miR-628-5p/AEG-1 axis. Liu et al [43]. showed that SNHG1 enhanced GC cell EMT progression via modulation of the miR-15b/Notch1/DCLK1 axis. Liu et al. proved that TONSL-AS1 modulated CDK1/miR-490-3p to regulate ovarian carcinoma cell growth [44]. Liu et al [45]. indicated that HNF1A-AS1 induced GC metastasis, angiogenesis, invasion and lymphangiogenesis by sponging miR-30b-3p. Shi et al [46]. demonstrated that OIP5-AS1 induced hepatocellular carcinoma angiogenesis, cell migration and cell growth by modulating miR-3163/VEGFA. Furthermore, Zhang et al. showed that knockdown of MSC-AS1 induced sensitivity to cisplatin and suppressed the development of osteosarcoma by sponging miR-142 [29]. We also showed that MSC-AS1 overexpression inhibited miR-142-5p expression in HGC-27 cells. We observed that DDK5 was a target gene of miR-142-5p. The overexpression of miR-142-5p suppressed the luciferase activity of wild-type DDX5, but the luciferase activity of mutant DDX5 was not changed. We showed that miR-142-5p was downregulated in GC specimens compared to paired normal specimens. MSC-AS1 expression was inversely correlated with miR-142-5p expression in GC specimens. MSC-AS1 induced cell growth, cell cycle progression and inflammatory mediator secretion by modulating DDX5.

In summary, we observed that MSC-AS1 was overexpressed in GC cells and specimens and that ectopic expression of MSC-AS1 enhanced cell growth, cell cycle progression and inflammatory mediator secretion by modulating miR-142-5p/DDX5. These results showed that MSC-AS1 acts as a key oncogene in the development of GC.

## MATERIALS AND METHODS

### Clinical specimens and cell transfection

A total of forty pairs of GC specimens and control specimens were acquired from GC patients who underwent surgery at The Fourth Hospital of China Medical University (Liaoning, Shengyang). Cell lines (HGC-27, MKN-45, SGC-7901, MGC-803 and GES cells) were acquired from ATCC, USA and cultured in DMEM (Gibco, BRL, UK) supplemented with streptomycin/penicillin and FBS (Gibco, BRL, UK). miR-142-5p mimic, pcDNA-MSC-AS1, siRNA-DDX5 and their controls (20 nM) were obtained from Shanghai GenePharma. Cell transfections were carried out with a Lipofectamine kit (Invitrogen, CA, USA).

### RT-qPCR

RNA was extracted from GC cells or specimens using a TRIzol kit following the manufacturer’s protocol (Invitrogen, CA, USA). We utilized SYBR Green reagent (NEWBio) to study miR-142-5p, DDX5 and MSC-AS1 expression with the CFX96 system (VisonBio Scientific). The data were normalized to GAPDH or U6. The 2^-ΔΔCt^ method was carried out to calculate relative fold changes. Primer sequences were noted: MSC-AS1, F, TCAAG AAATG GTGGC TAT and R, GCTCT GAGAC TGGCT GAA; miR-142-5p, F, TCAAG AAATG GTGGC TAT and R, CATAA AGTAG AAAGC ACTACT; U6, F, GCTTC GGCAG CACAT ATACT AAAAT and R, CGCTT CACGA ATTTG CGTGT CAT; GAPDH, F, GTCAA CGGAT TTGGT CTGTA TT and R, AGTCT TCTGG GTGGC AGTGAT.

### Luciferase assays

The DDX5 3’-UTR and mutated DDX5 3’-UTR were cloned into the pGL3 plasmid as wild-type or mutant type 3’-UTRs, respectively. GC cells were treated with the wild-type or mutant DDX5 3’-UTR together with miR-142-5p scramble or mimic using a Lipofectamine kit. After transfection for 2 days, the luciferase activity was determined by Dual-Glo luciferase analysis (Promega, WI, USA).

### CCK-8 assay, cell cycle analysis and ELISA

Cell growth was detected with a CCK-8 assay kit (Dojindo, Japan) according to the manufacturer’s instructions. These cells were plated in 96-well plates and cultured for 0, 24, 48 and 72 hours. Ten microliters of CCK-8 were added to each well, and the cells were cultured for an additional 3 hours. The absorbance was detected at 450 nm at different time points. To analyze cell cycle progression, GC cells were stained with cell cycle reagent (Thermo) following a standard protocol. The cell cycle was measured with flow cytometry on a Beckman flow cytometer (Dickinson, USA). The protein levels of IL-1β, IL-6 and TNF-α in the cell suspension were detected by ELISA following the manufacturer’s protocol.

### RNA Immunoprecipitation (RIP) analysis

The RIP assay was conducted utilizing the Magna RNA-Binding Protein Immunoprecipitation of the RIP Kit (Millipore) following standard instructions. The cells were harvested and then lysed in RIP lysis buffer containing RNase and protease inhibitor, and then, the lysates were treated for 2 hours with buffer containing magnetic beads coated with antibodies against Ago2. IgG served as the negative control. Coprecipitated RNAs were determined by RT-qPCR assay.

### Statistical analysis

All the statistical assays were analyzed using SPSS 19.0 (Chicago, IL, USA), and the graphs were generated by Prism 5.0. Student’s t-test was used to compare significant differences between two groups, and the correlation between miR-142-5p and MSC-AS1 in GC was analyzed by Pearson correlation assay. p<0.05 was defined as statistically significant.
